# PA28αβ: The Enigmatic Magic Ring of the Proteasome?

**DOI:** 10.3390/biom4020566

**Published:** 2014-06-19

**Authors:** Paolo Cascio

**Affiliations:** Department of Veterinary Sciences, University of Turin, Grugliasco 10095, Italy; E-Mail: paolo.cascio@unito.it; Tel.: +39-011-670-9113; Fax: +39-011-670-9138

**Keywords:** PA28αβ, proteasomes, immunoproteasomes, protein degradation, MHC class I antigen presentation, epitopes, antigenic peptides

## Abstract

PA28αβ is a γ-interferon-induced 11S complex that associates with the ends of the 20S proteasome and stimulates *in vitro* breakdown of small peptide substrates, but not proteins or ubiquitin-conjugated proteins. In cells, PA28 also exists in larger complexes along with the 19S particle, which allows ATP-dependent degradation of proteins; although *in vivo* a large fraction of PA28 is present as PA28αβ-20S particles whose exact biological functions are largely unknown. Although several lines of evidence strongly indicate that PA28αβ plays a role in MHC class I antigen presentation, the exact molecular mechanisms of this activity are still poorly understood. Herein, we review current knowledge about the biochemical and biological properties of PA28αβ and discuss recent findings concerning its role in modifying the spectrum of proteasome’s peptide products, which are important to better understand the molecular mechanisms and biological consequences of PA28αβ activity.

## 1. MHC Class I Antigen Presentation

The continual presentation of intracellular proteins fragments on major histocompatibility complex (MHC) class I molecules is a process that allows cytotoxic CD8^+^ T lymphocytes (CTLs) to identify and selectively eliminate cells that synthesize foreign (e.g., viral) or abnormal (e.g., oncogene products) proteins [1,2]. The vast majority of MHC class I-presented peptides (known as antigenic peptides or epitopes) are generated during the degradation of mature proteins or defective ribosomal products (DRiPs) by the ubiquitin-proteasome system (UPS) [3,4]. These peptides are then translocated through TAP transporters [5] to the endoplasmic reticulum (ER) where they bind to MHC class I heterodimers and are delivered to the cell surface [6]. The active form of the proteasome, which appears to degrade most cellular proteins, is the 26S proteasome [7,8]. This large (2.4 MDa) and abundant multi-subunit proteolytic complex consists of the 20S proteasome, in which proteins are degraded, capped at one or both ends by the 19S regulatory particle, which is responsible for recognizing, unfolding, and translocating polyubiquitinated (and some non-ubiquitinated, e.g., denatured) substrates into the 20S internal proteolytic cavity [9]. The 20S proteasome is a barrel-shaped structure composed of four stacked heptameric rings. The two outer rings consist of α-subunits, while the two central rings are made up of β-subunits [10]. In most cells, the proteolytic activity of the 20S proteasome is located at subunits β5 (X), β2 (Z), and β1 (Y) of the core particle. However, lymphoid cells and cells exposed to cytokines such as IFN-γ alternatively express three homologous subunits (β5i/LMP7, β2i/MECL-1, β1i/LMP2), which replace the constitutive ones in newly assembled so-called immunoproteasome particles [11]. A variety of studies have demonstrated that incorporation of these INF-γ-induced subunits quantitatively modifies proteasomal cleavage preferences and enhances the production of a significant number of antigenic peptides, although there are examples of epitopes that are generated with lower efficiency, or which are not released, by immunoproteasomes [12]. In any case, the recent generation of transgenic mice lacking all three proteasomal catalytic β-immune subunits clearly demonstrated the pivotal role that immunoproteasomes play in the generation of MHC class I ligands [13]. Additionally, immunoproteasomes have been shown to be important for efficient cytokine production [14] and have been implicated in a number of pathological disorders such as cancer and neurodegenerative and autoimmune diseases [15,16,17]. Finally, it has been recently demonstrated that 26S immunoproteasomes possess the capacity to hydrolyze basic proteins (such as Histones and Myelin Basic Protein) at greatly increased rates compared to constitutive proteasomes [18].

## 2. Proteasome Activator PA28αβ

### 2.1. Structure

Another INF-γ-inducible UPS component that affects MHC class I antigen presentation is PA28 (also termed REG or 11S), a ring-shaped 180 kDa multimeric complex that binds, in an ATP-independent manner, to the two ends of the 20S proteasome and substantially enhances its ability to degrade short peptide substrates, but not proteins or ubiquitin-conjugated proteins [19,20]. In addition, PA28 can also associate with the free end of asymmetric 26S proteasomes (19S-20S) to form so-called “hybrid proteasomes” (19S-20S-PA28) [21,22,23,24] that hydrolyze tri- and tetra-peptides at higher rates than canonical 26S particles [21,23]. In mammals, PA28 is composed of two homologous subunits, namely PA28α (REGα or PSME1) and PA28β (REGβ or PSME2), both of which are induced by γ-interferon [25,26,27,28,29,30,31]. A third member of PA28 family is PA28γ (also known as REGγ, 11Sγ or Ki antigen), which is a nuclear antigen that is not induced by INF-γ and not involved in MHC class I antigen presentation. The biochemical and biological properties of PA28γ has been reviewed elsewhere [32], and consequently will not be considered in detail.

PA28α and PA28β have an apparent molecular weight of 28 kDa on SDS-PAGE electrophoresis and share a nearly 50% amino acid sequence identity [25,29,33]. Based on the crystal structure of PA28α [34], the overall secondary structure of PA28 proteins is composed of four long α-helices of 33–45 residues in length that are involved in intra- and intermolecular interactions. The linker sequence between helices 2 and 3, which is highly conserved in PA28 α and β subunits, is designated the “activation loop” since it is responsible for stimulation of proteasome peptidase activities [35]. A second region of the molecule involved in the activation of 20S proteasome is the 10 residue C-terminal tail, which provides binding energy for PA28-proteasome association [36,37,38]. Finally, the linker between helices 1 and 2 is composed of sequences that are highly divergent between PA28 α and β subunits and for this reason are known as “homolog specific inserts” [39]. Although these inserts are not resolved in the X-ray structure of PA28α, presumably since they are flexible, it is almost certain that they protrude from the upper surface of the PA28α ring [30].

PA28 has been reported to be phosphorylated *in vivo* on serine residues [40,41] and phosphorylation was believed to be indispensable to activate proteasome peptidase activities [41]. Considering, however, that nearly all *in vitro* studies concerning the biochemical properties of PA28 have been performed using the recombinant protein expressed in *E. coli* (which in the vast majority of cases are not competent for phosphorylation of recombinant proteins), it is clear that phosphorylation is not essential for the stimulatory activity of PA28, while *in vivo* it may exert more subtle and yet unidentified regulatory functions. Moreover, binding of PA28 to calcium has also been described although the physiological significance of this observation remains an open question [42].

While it is clear that at least *in vitro* recombinant PA28α can form a heptameric ring [34,43] and recombinant PA28β is a monomer [35,39], the subunit stoichiometry of native PA28αβ has been initially reported [26,38] to be a hexamer (3α3β). However, subsequent studies indicated that heteromeric PA28 is a heptamer formed by the association of three α subunits and four β subunits [44,45]. Electron microscopy images show that PA28αβ forms a cap on the end of 20S particle by interacting with proteasome α-subunits [46]. This cap is about 10–11 nm wide at the base, where it attaches to proteasome α subunits, and 7–8 nm long from the base to the tip. EM data also indicate that PA28 contains a central channel that apparently traverses it entirely to the central pore of proteasome α-ring. Accordingly, the crystal structure of recombinant PA28α at 2.8 Å resolution reveals a heptameric ring traversed by a central aqueous channel with a diameter of 30 Å on the face contacting the proteasome and 20 Å on the other [34].

### 2.2. Role in MHC Class I Antigen Presentation

The effects of PA28αβ on antigen processing and CD8^+^ T-cell responses are still unclear and controversial [2,30]. Professional antigen presenting cells generally express PA28αβ at high levels, which is in agreement with a possible function of this complex in MHC class I antigen processing [47,48]. Accordingly, expression of PA28α alone [49,50] or PA28αβ [50,51,52,53,54] has been reported to enhance MHC class I-presentation of some, but not all, antigens. Furthermore, cells lacking this complex have a reduced ability to generate certain antigens [55], and recent studies identified the second most important UPS component in PA28 (surpassed only by β5i/LMP7) for production of MHC class I ligands [56], although its effects seem to be restricted to specific MHC class I alleles [57].

Although concerted expression of PA28 α and β and proteasomal β1i, β2i and β5i subunits following INF-γ induction leads to the formation of PA28αβ-20S immunoproteasomes *in vitro* and *in vivo* [22,24,26,31,55,58,59,60,61,62,63,64], however PA28αβ can also be detected in cells, tissues and organs that lack immunoproteasomes like erythrocytes, muscle and brain [19,20,58,59,60,61,65,66]. It is, therefore, conceivable that PA28αβ can exert its biological function(s) also in association with constitutive proteasomes, probably related to its capacity to substantially modify the pattern of proteasomal peptides products. Further studies will be required to assess whether the changes in the patterns of peptides generated from proteins following binding of PA28αβ with 20S and 26S constitutive are similar to those already identified for its association with 20S and 26S immunoproteasomes [18].

### 2.3. Biochemical Properties

#### 2.3.1. Hydrolysis of Fluorogenic Peptide Substrates

**Figure 1:**
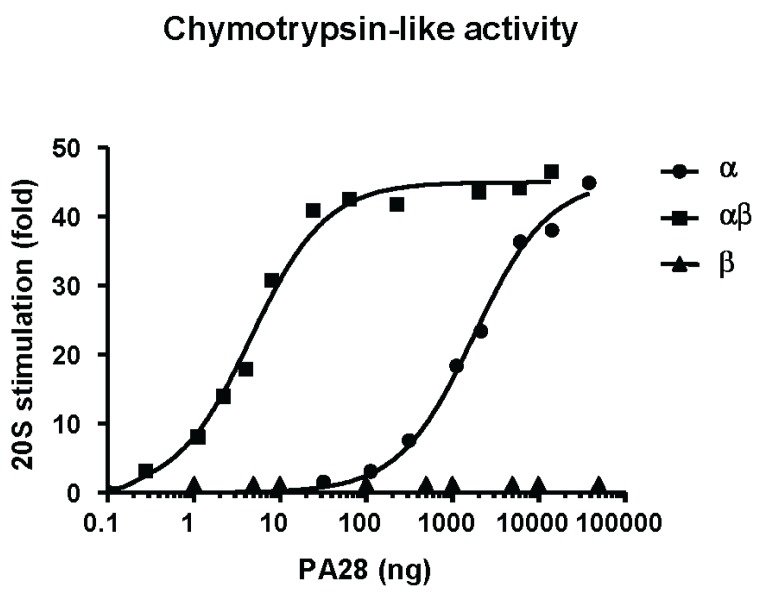
Effect of PA28α, PA28αβ and PA28β on the chymotrypsin-like activity of 20S immunoproteasome. Assays were carried out *in continuo* at 37 °C in 500 μL of buffer reaction 20 mM Tris-HCl pH 7.5, 0.2% (w/v) BSA, containing 100 μM of Suc-LLVY-AMC. For each assay, 4 ng of 20S were used.

As pointed out above, PA28αβ was originally identified due to its capability to markedly stimulate peptidase activities of the 20S proteasome, as measured using short fluorogenic model substrates. In particular, kinetic studies revealed that PA28 increases *V*_max_ and reduces *K*_m_ for peptide hydrolysis by proteasomes [19,20]. Subsequent detailed biochemical analysis demonstrated that the native and recombinant PA28α subunit is able to activate all three main proteasomal peptidase activities [35,39,65,67]. Nonetheless, the precise biochemical functions of PA28β are still unclear and a matter of debate. While it is well established that, by association with the α subunit, PA28β strongly enhances the affinity for the proteasome of the PA28 heterocomplex [38,65,67], whether the β subunit by itself activates the 20S particle is controversial, as acknowledged in several studies [35,36,39,52], but excluded in others [31,65,67]. Paradoxically, in one investigation PA28β was reported to activate proteasomes at high concentrations, but behaves as an allosteric proteasome inhibitor at low concentrations [68]. To investigate this issue, we checked the effect of our standard preparation of recombinant PA28β on proteasomal chymotryptic activity. From our experiments, it is clear that PA28β is able to associate with the α subunit and to greatly increase its affinity for the 20S proteasome, but by itself it does not exert any stimulatory effect on proteasome chymotrypsin-like activity (Figure 1).

Concerning the exact molecular mechanisms of proteasome activation by PA28, a variety of biochemical actions have been proposed, including allosteric modification of the 20S proteasome active sites [20,69] or non-catalytic modifier sites [69], stimulation of peptide entry into the particle [20,52,70], stimulation of peptide exit [52,71], and facilitating the binding of proteasomes to chaperones or to components of the endoplasmic reticulum [30]. In particular, PA28 has been often proposed to enhance hydrolysis of short peptides by inducing long-range conformational changes in proteasomal active sites. In fact, biochemical data indicates that proteasome proteolytic sites are allosterically regulated [36,72,73,74,75] and that their modification leads to gate opening [76,77,78]. Furthermore, an allosteric pathway linking the PA26 (the PA28 homologue in trypanosomes) binding sites with the active sites in the *T. acidophilum* 20S proteasome has been recently described [79]. On the other hand, crystallographic studies unambiguously showed that association of yeast 20S with PA26 does not induce any structural modification of proteasomal catalytic β subunits [71], thus disproving an allosteric mechanism for its stimulatory effect. It remains, however, possible that subtle changes in the β-rings of 20S proteasomes induced by PA28 are lost in the rigid crystal structure of PA26-20S but might be detectable with other techniques (e.g., NMR or EM) [80,81]. PA28 has also been reported to enhance the ability of 20S proteasomes to make correct C- and N-terminal cleavages in longer oligopeptides *in vitro* in order to generate class I presented epitopes [82,83]. However, other studies indicate that proteasomes generate N-extended precursors of antigenic peptides [84] that are trimmed to their presented epitopes by cellular aminopeptidases [85,86,87,88,89,90]; this N-terminal trimming is also stimulated by γ-interferon [86,90,91], thus strongly questioning the *in vivo* relevance of the double cut model. Lastly, PA28 was reported [92] to be essential for assembly of immunoproteasomes, but subsequent studies failed to confirm this finding [55,93].

#### 2.3.2. Gate Opening Mechanism

In the crystal structure of PA26 solved by Hill and coworkers [71], the binding of PA26 was found to open the gate on the channel in the proteasome α-ring through which substrates enter [94] and products exit [70]. Specifically, PA26 C-terminal residues dock into pockets between adjacent proteasome α subunits and, by forming hydrogen bonds and a salt bridge between the C-terminal carboxylate of the activator and a highly conserved proteasome lysine side chain (Lys 66), provide binding energy for PA26-20S complexes [71,95]. Binding to the C-termini of PA26, however, is not sufficient to activate the 20S proteasome, which requires the activation loop that forms a seven-fold symmetric circular array that interacts with the base of the N-terminal gating residues of the seven proteasomal α subunits. In particular, a glutamate side chain (Glu 102) in each PA26 subunit activation loop contacts and repositions a proline residue (Pro 17) of 20S α subunits located above the surface of the proteasome. This interaction triggers gate opening by disrupting packing and hydrogen bonding interactions of the asymmetrical closed conformation and by widening the pore opening to a more symmetrical arrangement that allows a belt of intersubunit contacts to form around the circumference of the opening [96,97].

On the basis of these structural observations, PA28 was predicted to lead to attenuation of proteasomal processivity and consequent release of peptide products of greater mean length [71], as occurs on deletion of the α-gate [70]. Since most peptides released by proteasomes are too short to bind to MHC class I molecules [84,98,99,100,101], the generation of larger products would be expected to enhance the fraction of products capable of serving in antigen presentation, either directly or after trimming by aminopeptidases in the cytosol [89,102] or endoplasmic reticulum [86,87,88,90,103]. However, since not only PA28 but also the 19S regulator can induce the opening of the 20S core particle central gate [104], it remains unclear whether the above-mentioned structural observations can explain the specific effects of PA28 on substrate cleavage and, consequently, on antigen presentation.

#### 2.3.3. Effects of PA28αβ on Protein Degradation

In this respect, it is clear that a full understanding of the specific biochemical functions of PA28αβ requires quantitative information on the rates of protein substrate hydrolysis and generation of peptide products by PA28αβ-containing immunoproteasomal species. To address this important issue, we recently performed a systematic analysis of the entire spectrum of peptides released during degradation of full-length proteins by PA28αβ-20S immunoproteasomes [101]. PA28αβ-20S particles were found to hydrolyze proteins at identical, slow rates compared to 20S proteasomes, and to generate higher amounts of very short products together with several longer peptides characterized by higher overall hydrophilicity.

The finding that unstructured polypeptides are hydrolyzed at rates that are nearly 10-fold higher by 26S compared to 20S and PA28αβ-20S immunoproteasomes is consistent with the notion that the free 20S particle is a relatively inactive protease since the N-terminal tails of its α subunits obstruct the two opposite axial pores through which substrates access the internal catalytic lumen [94]. This autoinhibited state is relieved when the 20S core particle binds to activators such as 19S or PA28 that displace, in an ATP-dependent and independent manner, respectively, the N-terminal tails thereby opening an axial channel in the α annulus [70,71]. However, the latency of unliganded 20S proteasome is not absolute since, even in the absence of artificial treatments (e.g., heating or presence of low concentrations of detergents or chaotropic agents) that are known to activate it [10], the 20S core particle degrades proteins at detectable and reproducible rates, probably involving transient and/or only partial channel opening [70,76,77,78]. Surprisingly, despite the fact that the open-channel conformation of the gating residues induced by ATP-dependent and independent activators appears to be identical [105], our data show that PA28αβ-20S immunocomplexes hydrolyze proteins at the same rates as 20S immunoproteasomes, but much less efficiently than 26S immunoproteasomes. While on one hand this confirms initial reports on the biochemical properties of PA28 [19,20,65], it is difficult to reconcile with the known role of the proteasomal gate in controlling accessibility of substrates into the lumen of the 20S particle. In fact, opening of the axial channel by deletion of the N-terminal tails of eukaryotic [70,106] or archaeal [107] proteasomal α subunits results in strong enhancement of the degradation rates of unfolded proteins, therefore suggesting that unstructured substrates can freely access the internal proteolytic chamber of the 20S particle simply by passive diffusion through a fully open gate. Furthermore, although it has been proposed that translocation of completely unstructured proteins might require a driving force produced by ATP hydrolysis [108], subsequent studies demonstrated that once the polypeptide chain is unfolded its transit through the ATPases ring can occur by passive diffusion, in which retrograde movement is probably prevented by a Brownian ratchet mechanism [109]. In light of these data, the inability of PA28αβ to enhance hydrolysis rates of loosely folded proteins is surprising. It can be speculated that the presence of a supplementary multimeric ring, sitting at the proteasomal outer α surface, might impose an extra constraint to the completely free diffusion of large, although linear, polypeptide chains into the internal proteasomal lumen. From this point of view, transit thorough the pore of open gate mutants might not perfectly reflect passage across the PA26/28 central channel. In fact, the crystal structure of PA28α shows that the aqueous channel through the heptamer has a diameter of 20 Å at its minimum, which is theoretically wide enough for passage of unfolded proteins [34]. However, the homolog-specific inserts present between helices 1 and 2, which are not resolved in the crystal structure, most likely form a ring-like collar on the upper, non-proteasome binding surface of the PA28 heptamer. Although several studies have shown that these loops do not restrict passage of tri- or tetra-peptide fluorogenic substrates [35,67], recent investigations have demonstrated that they can hinder the transit of longer peptides, and conceivably of proteins as well, through the PA28 channel [44]. In fact, we demonstrated that 20S and PA28αβ-20S immunoproteasomes hydrolyze proteins at exactly the same slow rates, which implies that denatured substrates transit through the partially or transiently open gate of unligated 20S and the fully open channel of PA28αβ-20S particles with comparable efficiency. However, it cannot be excluded that PA28αβ might selectively enhance degradation of some specific substrates that are yet to be identified, as has been unambiguously demonstrated for PA28γ [110,111,112,113].

#### 2.3.4. Effects of PA28αβ on Peptide Products Generation

Although PA28αβ is unable to enhance rates of protein degradation by proteasomes, its association with the 20S particle was found to lead to substantial changes in the patterns of peptides generated, which greatly differ from those produced by 20S and 26S immunoproteasomes [101]. In fact, only ~10% of peptides generated by 20S and 26S immunoproteasomes were found to be 8–10 residues long [101], which is the appropriate length to bind MHC class I heterodimers. Most importantly, association of PA28αβ with the ends of 20S immunoproteasomes does not increase the fraction of 8–10 residue peptides generated, but reduces it to 6% of the total. Moreover, the fraction of peptides longer than 10 amino acids, which might serve in MHC class I antigen presentation only after appropriate trimming by aminopeptidases in the cytosol or ER, is larger for 20S than for 26S immunoproteasomes; binding of PA28αβ to the 20S particle dramatically reduces the overall efficiency of generation of these longer products. It is thus clear that PA28 does not act simply by expending the fraction of proteasomal products that can be accommodated in the groove of MHC class I molecules directly or after trimming. In this regard, it is worth noting that both 20S and 26S immunoproteasomes display a similar high propensity to release 8–10 residue long products, although 20S has also an increased capacity to generate longer fragments [101]. However, correct evaluation of the overall efficiency of different immunoproteasome species in producing peptides with a size potentially suitable for class I antigen presentation requires normalization of their rates of generation with those of substrate hydrolysis. Therefore, since *in vitro* 26S immunoproteasomes were found to degrade unfolded proteins at 10-fold higher rates than 20S and PA28αβ-20S [84,101,114], it is evident that 26S holoenzymes are potentially the most efficient immunoproteasomal species in terms of generating higher amounts of peptides with the correct size to serve in MHC class I antigen presentation either directly or after aminopeptidase trimming.

#### 2.3.5. PA28 as a Smart Sieve

Surprisingly, PA28αβ-20S immunoproteasomes were found to display a reduced ability to generate longer products that, in principle, might depend upon conformational changes in proteasomal active sites. This hypothesis has already been suggested to explain the biochemical properties of PA28 [36,72,75], and specifically its ability to stimulate coordinated dual cleavages of short synthetic peptides (typically 19–25 residues long) by 20S particles [82,83]. This possibility, however, seems unlikely for PA28αβ-20S immunoproteasomes since, as already pointed out, association with PA26 does not induce any structural modification of proteasomal catalytic β subunits [71]. Alternatively, PA28αβ might primarily act as a molecular sieve that retains longer protein fragments inside the 20S proteolytic chamber until they are cleaved to peptides that are small enough to diffuse to the outside. This later model would be consistent with detailed kinetic analyses showing that PA28 exerts its activating influence by enhancing bi-directional passage of short (3–4 residues) peptides [52] and with an important *in vitro*/*in silico* study that identified one of the major factors involved in the enhancement of double cut efficiency induced by PA28 in a reduced efflux of longer peptides out of the 20S particle [115]. Furthermore, it was recently shown that a PA28αβ complex lacking the unstructured and highly mobile PA28α loops surrounding the central pore of the heptameric ring cleaves substrates longer than a nonpeptide more efficiently than wild type PA28. On these bases, it was hypothesized that the flexible loops of PA28 might act as gatekeepers that block the exit of longer peptides from the proteolytic chamber [44]. Selectivity based exclusively on peptide size, however, cannot account for the overall effects of PA28 on the patterns of proteasome products observed by Raule *et al.* [101]. In fact, quantitation of products demonstrated that several individual peptides with a length of 8–23 residues are released in much higher amounts by PA28αβ-20S than by 20S or 26S immunoproteasomes [101]. Therefore, PA28 appears to act as a selective filter that promotes preferential passage of only subset of specific long products through its central channel, presumably based on sequence.

**Figure 2:**
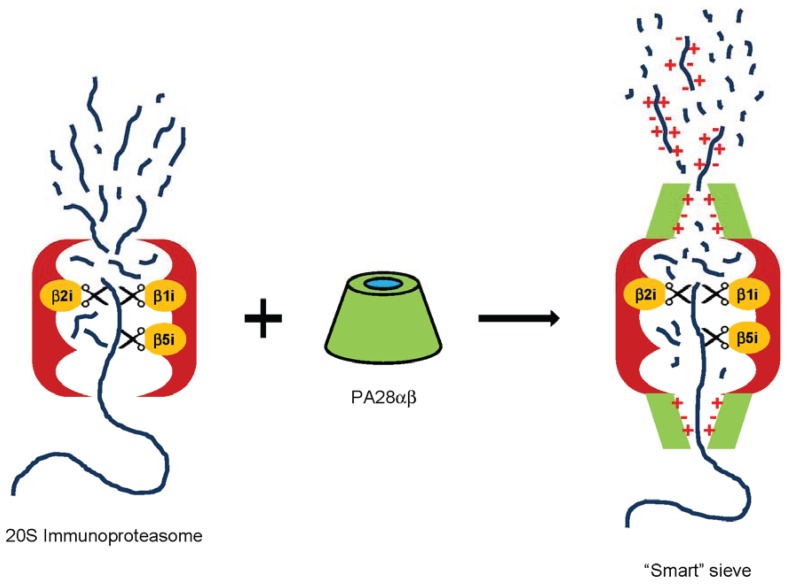
“Smart” sieve model for biochemical activity of PA28αβ. See text for more details.

At present, the properties that might allow specific longer peptides to evade the constraint imposed by PA28 towards their efflux are not completely clear. However, the finding that products longer than seven residues whose generation is strongly enhanced in the presence of PA28 are, on average, more hydrophilic than those preferentially released by 20S alone [101], strongly suggests that the passage of polar/charged long peptides thorough PA28 might be favored. In this model, PA28 would act as a selective “smart” sieve that strictly controls the exit of products from proteasomes on the basis of size and sequence (Figure 2). As a result, PA28αβ would promote preferential efflux from the 20S proteolytic cavity of only a reduced number of individual peptides longer than 6–7 amino acids, while the majority of the other proteasomal products are retained inside where they are further cleaved to smaller pieces before they diffuse outside. In accordance with this model, the central channel of PA28α ring is almost completely lined by charged or polar residues [34], and is thus well suited for permitting the passage of water soluble peptides. Importantly, this molecular model would be also consistent with our findings on 19S-20S-PA28 immunoproteasomes. In this case, the absence of a clear difference in size distribution [21] argues that in hybrid proteasomes (as in 26S canonical particles) the main route of exit of peptides from the inner proteolytic chamber is regulated by the 19S cap, while PA28 would exert its major effect by allowing preferential sorting of selected products through its central channel.

### 2.4. Other Potential Biological Functions

A full understanding of the exact biological functions of PA28 would undoubtedly represent an important achievement, especially in the light of the observation that mammalian cells contain significant amounts of PA28αβ-20S immunocomplexes [22,24,26,58,59,60,61] whose abundance further increases upon INF-γ stimulation [31,55,62]. In this respect, it seems extremely unlikely that PA28 functions *in vivo* to stimulate degradation of cytosolic oligopeptides by proteasomes, because such peptides are very rapidly hydrolyzed by other cellular exo- and endo-peptidases [116]. Moreover, it has been shown *in vitro* that peptides released by proteasomes are further cleaved by these proteolytic particles at extremely low rates [117], and they are thus unlikely to efficiently compete for degradation with proteins, which are much more preferred substrates [118].

Importantly, the advantage of PA28-containing proteasomes that generate highly divergent patterns of potential antigenic peptides, characterized by profound qualitative and quantitative differences, might become relevant under specific pathophysiological conditions especially if favoring a non-canonical, ubiquitin-independent, protein hydrolysis pathway. In fact, most of the *in vitro* studies addressing the production of antigenic peptides have focused on degradation by proteasomes of denatured, non-ubiquitinated proteins since ubiquitinated proteins are not hydrolyzed by 20S and PA28-20S complexes [10,19]. This is likely due to the fact that these particles lack the enzymatic activities necessary to remove and/or to unfold polyubiquitin chains that otherwise would sterically block translocation of substrates into the proteolytic chamber [119,120,121]. Although further studies will be required to clarify whether the patterns of peptides released are different if a protein substrate initially binds to the 19S regulatory particle through a polyubiquitin chain or directly through an unstructured domain, several lines of evidence indicate that *in vivo* denatured proteins are important, physiological substrates for intracellular proteolysis. A large fraction of MHC class I epitopes is derived from rapid degradation of DriPs [122,123,124] in a process that has been shown to be at least partially ubiquitin-independent [124,125,126]. Furthermore, several recent reports indicate that some loosely-folded, short-lived regulatory, viral and oxidized proteins are degraded *in vivo* by the 20S proteasome in an ATP- and ubiquitin-independent manner [126,127,128,129,130,131,132,133]. In this regard, a contribution of PA28 in this alternative pathway of protein catabolism pathway seems plausible. Moreover, immunoproteasomes were recently reported to be strongly induced under conditions of oxidative stress [134], and according to several lines of evidence, oxidized proteins are preferentially degraded without ubiquitination by 20S proteasomes [131] in a process that was reported to be stimulated by PA28αβ [135,136,137]. Importantly, oxidative damage of DNA is recognized as an important cause of malignant transformation and cancer development [138], which emphasizes the importance of efficient MHC class I immune surveillance in the presence of oxidative stress. Of note, PA28αβ was shown to be highly induced in a naturally occurring tumor [15]. Under conditions of altered redox homeostasis, therefore, the pool of peptides specifically or preferentially released by PA28αβ-20S immunoproteasomes might be critical in eliciting an effective CTL response. Furthermore, by enhancing fragmentation of the large majority of proteasomal products, but at the same time, by promoting release from the 20S particle of specific peptides with a length of eight or more residues, PA28 is likely to exert a profound influence on the immunodominance hierarchy of CD8^+^ responses. Finally, it is also conceivable that by promoting release of peptides that apparently cannot serve in class I antigen presentation [101], PA28 might exert a regulatory function aimed at blunting excessive cytotoxic responses against antigens of self-origin, thus preventing the risk of potentially harmful autoimmune reactions.

## 3. Conclusions

Although PA28 has been discovered more than 20 years ago, its precise biological functions have remained somewhat elusive, and despite that several *in vitro* and *in vivo* studies have attempted to clarify its effects on MHC class I antigen presentation pathway, its role in adaptive immunity is still quite unclear. Recent data, however, indicate that the main function of PA28 may be related to its capacity to enhance generation by immunoproteasomes of subset of specific more hydrophilic peptides. Further studies will be required to unveil the specific role of these hydrophilic products and to clarify whether they are favoured in some steps of the MHC class I antigen processing pathway.
